# Supplementary material for: Modelling vegetation land fragmentation in urban areas of Western Province, Sri Lanka using an Artificial Intelligence-based simulation technique
Source: PLoS One. 2023 Feb 6;18(2):e0275457. doi: 10.1371/journal.pone.0275457 (PMC9901792; doi:10.1371/journal.pone.0275457)
Supplement: Attachment — Submitted filename: Recommendations.docx [file pone.0275457.s002.docx]

As I read the manuscript carefully, I found some areas in which I would have appreciated greater clarity. I believe the paper could be further strengthened by changes which should be made before it gets published as follows:

**The title**

As the title of any paper should attract the reader and concisely reflects the actual content of the researchers’ contribution, I found the title of this manuscript needs some modification. For example, I suggest the following title “***Modelling vegetation land fragmentation in urban areas of Western Province, Sri Lanka using an artificial intelligence-based simulation technique***”

**The Abstract**

While the abstract summaries the main idea of fragmentation as a landscape ecology term properly, several keys are missing from it mainly the research aims. Please directly state the main aim and objectives (or the research questions) of the study and write about the policy implications of the study area. Likewise, I suggest inserting a couple of sentences to mention all variables that have been incorporated in the modelling process.

**Introduction**

Line 44 what does the number 392249 refer to and it seems that the citation is written in an integrated or standard way?

Line 62-63 the sentence needs to rewritten.

Line 64 the authors have mentioned “studies point to….” while they have cited only one study (32).

Overall, the introduction section involves both a theoretical framework and related work as well as introducing the main idea of the research. Nevertheless, it was NOT written in cohesion and logical flow style. Similarly, the analytical procedures, utilized spatial techniques for the analysis of land use land cover dynamics have not been made clear.

**Theoretical Framework**

Besides the poor scientific literature about fragmentation of lands, spatial patterns and ecological process, the literature review section also lacks an adequate description of methods and analyses that previously used in studying several land use land cover LULC applications particularly outside the study area and region. I suggest, to expand the literature review by including additional references particularly the following:

- **Spatial disparity patterns of green spaces and buildings in arid urban areas**
- **Cultivated Land Fragmentation and Its Influencing Factors Detection: A Case Study in Huaihe River Basin, China**
- **The evolution of urban sprawl: Evidence of spatial heterogeneity and increasing land fragmentation**

Other issues in this section should be resolved as follows:

- The authors should combine the two sections “the introduction and theoretical framework” into one section entitled “***introduction and literature review***”.
- The authors should explain to the reader what motivated this research and why it is important to assess the vegetation land fragmentation in this area specifically.
- Similarly, and from a methodological perspective, various questions should be answered such as how the previous research analyzed and investigated vegetation land fragmentation in Sir Lanka and outside? What are the main spatial methods and techniques that have been used?
- The literature review is very poor and must be extended to include several studies that cover various issues that are relevant to this research.

**Methods**

The title “method” should be changed into “material and methods”.

I am wondering why the authors have not represented the identified parameters in a map. The authors should provide the readers with a map illustrates all spatial variables utilized in the analysis processes.

Line 187 the explanation of the equation’s symbols should be placed after NOT before.

**Findings**

The authors should represent the spatial outputs of the modelling process. For instance, the reader expects to see one map for each category such as corridor, patch, and matrix.

The authors should add at least two maps for more clarity of the finding’s representation.

**Figure 12.** “Rules of variables according to lekprofile algorithm” should be reproduced in a higher level of resolution.

Overall, the results section lacks any sort of cause-and-effect relationships investigation. Consequently, the results have been written in methodological way and thus the authors must modify this section based on spatial patterns distribution across the study area.

**Discussion and conclusions**

The discussion should tell the story of the science and answer the “why” question of the results.

The explanation is not adequately supported by pieces of evidence, reasoning, and thus, ***after carrying out the suggested analyses***, a major revision is required for this section to clarify the discussion part and link it to the previous research.

The authors should mention the study’s limitations and drawbacks.

Many grammatical, stylistic and syntax errors are found across the manuscript.
