# Supplementary material for: Modelling vegetation land fragmentation in urban areas of Western Province, Sri Lanka using an Artificial Intelligence-based simulation technique
Source: PLoS One. 2023 Feb 6;18(2):e0275457. doi: 10.1371/journal.pone.0275457 (PMC9901792; doi:10.1371/journal.pone.0275457)
Supplement: Attachment — Submitted filename: Response to Reviewers.docx [file pone.0275457.s003.docx]

**Response to Reviewers**

Dear Editor,

We would like to thank the Journal of PLOS ONE for giving us the opportunity to revise Manuscript ID: PONE-D-21-37017. We thank the reviewers for their constructive comments. We have carefully taken their comments into consideration in preparing our revision. Below is our response to their comments.

Thanks for all comments.

Best wishes,

Amila Jayasinghe

**Response to Editor/Journal Requirements**

1.Please ensure that your manuscript meets PLOS ONE's style requirements, including those for file naming.

**Response:** We have rearranged the manuscript according to the PLOS ONE's style requirements.

2. We suggest you thoroughly copyedit your manuscript for language usage, spelling, and grammar. If you do not know anyone who can help you do this, you may wish to consider employing a professional scientific editing service.

**Response:** We have employed a professional scientific editing service. The country’s current economic crisis and restrictions on international money transfer prevent authors to obtain international services for language editing. However, if reviewers feel further the importance of editing language, we would like to do it before the final submission.

Name of the colleague: Shereen Rodrigo

Email: [srodriggo@gmail.com](mailto:srodriggo@gmail.com)

The name of the colleague or the details of the professional service that edited your manuscript

A copy of your manuscript showing your changes by either highlighting them or using track changes (uploaded as a *supporting information* file)

A clean copy of the edited manuscript (uploaded as the new *manuscript* file)

3. We note that the grant information you provided in the ‘Funding Information’ and ‘Financial Disclosure’ sections do not match.

When you resubmit, please ensure that you provide the correct grant numbers for the awards you received for your study in the ‘Funding Information’ section.

**Response:** We have included it in the submission form.

4. Thank you for stating the following financial disclosure:

“NO - Include this sentence at the end of your statement: The funders had no role in study design, data collection and analysis, decision to publish, or preparation of the manuscript.”

At this time, please address the following queries:

a)	Please clarify the sources of funding (financial or material support) for your study. List the grants or organizations that supported your study, including funding received from your institution.

b)	State what role the funders took in the study. If the funders had no role in your study, please state: “The funders had no role in study design, data collection and analysis, decision to publish, or preparation of the manuscript.”

c)	If any authors received a salary from any of your funders, please state which authors and which funders.

d)	If you did not receive any funding for this study, please state: “The authors received no specific funding for this work.”

Please include your amended statements within your cover letter; we will change the online submission form on your behalf.

**Response:** We have included it in the submission form.

5. Thank you for stating the following in the Acknowledgments Section of your manuscript:

“The authors would like to acknowledge the Senate Research Committee (SRC) Grant, University of Moratuwa, Sri Lanka (No SRC/ST/2021/XXX).”

We note that you have provided additional information within the Acknowledgements Section that is not currently declared in your Funding Statement. Please note that funding information should not appear in the Acknowledgments section or other areas of your manuscript. We will only publish funding information present in the Funding Statement section of the online submission form.

Please remove any funding-related text from the manuscript and let us know how you would like to update your Funding Statement. Currently, your Funding Statement reads as follows:

**Response:** We have removed it from the manuscript.

“NO - Include this sentence at the end of your statement: The funders had no role in study design, data collection and analysis, decision to publish, or preparation of the manuscript.”

Please include your amended statements within your cover letter; we will change the online submission form on your behalf.

**Response:** We have included it in the submission form.

6. In your Data Availability statement, you have not specified where the minimal data set underlying the results described in your manuscript can be found. PLOS defines a study's minimal data set as the underlying data used to reach the conclusions drawn in the manuscript and any additional data required to replicate the reported study findings in their entirety. All PLOS journals require that the minimal data set be made fully available.

Upon re-submitting your revised manuscript, please upload your study’s minimal underlying data set as either Supporting Information files or to a stable, public repository and include the relevant URLs, DOIs, or accession numbers within your revised cover letter.

**Response****:** We have shared the link of the study’s minimal underlying data set. Data (file://DESKTOP-EF7O048/Data)

7. We note that Figure 1, 2, 3 and 15 in your submission contain map images which may be copyrighted. All PLOS content is published under the Creative Commons Attribution License (CC BY 4.0), which means that the manuscript, images, and Supporting Information files will be freely available online, and any third party is permitted to access, download, copy, distribute, and use these materials in any way, even commercially, with proper attribution. For these reasons, we cannot publish previously copyrighted maps or satellite images created using proprietary data, such as Google software (Google Maps, Street View, and Earth).

We require you to either (1) present written permission from the copyright holder to publish these figures specifically under the CC BY 4.0 license, or (2) remove the figures from your submission.

**Response:** We have removed Figures 1, 2, 3 and 15 and supplied replacement figures.

8. We note you have included a table to which you do not refer in the text of your manuscript. Please ensure that you refer to Table 2 in your text; if accepted, production will need this reference to link the reader to the Table.

**Response:** We have changed it to Table 2.

**Reviewers' comments:**

1. Is the manuscript technically sound, and do the data support the conclusions?

The manuscript must describe a technically sound piece of scientific research with data that supports the conclusions. Experiments must have been conducted rigorously, with appropriate controls, replication, and sample sizes. The conclusions must be drawn appropriately based on the data presented.

**Response:** We have improved the manuscript as per the reviewers' comments.

2. Has the statistical analysis been performed appropriately and rigorously?

**Response:** We have improved the manuscript as per the reviewers' comments.

3. Have the authors made all data underlying the findings in their manuscript fully available?

The PLOS Data policy requires authors to make all data underlying the findings described in their manuscript fully available without restriction, with rare exception (please refer to the Data Availability Statement in the manuscript PDF file). The data should be provided as part of the manuscript or its supporting information, or deposited to a public repository. For example, in addition to summary statistics, the data points behind means, medians and variance measures should be available. If there are restrictions on publicly sharing data—e.g. participant privacy or use of data from a third party—those must be specified.

**Response:** We have shared the link of the study’s minimal underlying data set. Data (file://DESKTOP-EF7O048/Data)

4. Is the manuscript presented in an intelligible fashion and written in standard English?

PLOS ONE does not copyedit accepted manuscripts, so the language in submitted articles must be clear, correct, and unambiguous. Any typographical or grammatical errors should be corrected at revision, so please note any specific errors here.

**Response:** We have employed a professional scientific editing service.

**Reviewer #1**

**Abstract**

There is a difference between vegetation fragmentation and land (landscape) fragmentation? Authors should stick to standard terminology of either vegetation fragmentation or land/landscape fragmentation. If they are interested in understand fragmentation of various land cover or land uses (built-up, wetlands, forests etc) then they should stick to landscape or land fragmentation. If they are interested in the fragmentation of grasslands, forests and other green spaces they should stick to vegetation fragmentation**.**

**Response:** We have explained vegetation fragmentation in the entire manuscript.

Why the authors jumped to mention that,’’ By addressing current research gaps, the objective of this study is to develop an AI-based simulation framework to simulate vegetation land fragmentation in urban areas’’ without specifically highlighting the specific research gap?

**Response:** The highlighting research gap is unavailability of AI-based simulation framework to simulate vegetation land fragmentation. We have rearranged it by removing “By addressing current research gap”.

The authors should mention R as a statistical software ie R (statistical software) or R statistical software.

**Response:** We have changed it to statistical software.

**Introduction**

Please use the standard terminology and avoid ‘‘vegetation land-use changes’’. There is nothing called vegetation land-use change/s

**Response:** We have changed it to “vegetation cover changes”.

The use of numbering in citation should be consistent. In the number reference system, a number is normally added in parentheses or square brackets in the appropriate place in the text, starting the numbering from 1. Furthermore, the reference bibliography section of the research paper or manuscript is arranged by the order in which the citations appear in the text. Please check again and stick to journal guidelines.

**Response:** We have corrected the errors in citations and references.

Line 52-61, vegetation fragmentation or land fragmentation is poorly defined. It be should be brief, succinct and not long and winding especially in a journal article.

**Response:** We have clearly defined vegetation fragmentation as the division of vegetation patches into smaller ones.

Line 74-78, Again, this statement ‘‘Vegetation land fragmentation refers to variations in shape, size, composition, and distribution of vegetation. As a result, researchers use landscape metrics such as the division index, patch density, number of patches, area-weighted index, and others to evaluate vegetation land fragmentation [7][21]. As a result, it's critical to define vegetation land fragmentation before attempting to quantify..’’ is a repetition of the definition of vegetation or land fragmentation (ie Line 52-61).

**Response:** We have removed the repetitions and reordered the introduction section.

Line 111-119, the statement, ‘‘therefore, based on landscape ecology… The Patch-Corridor-Matrix model, which is the essential approach for quantifying vegetation land fragmentation in landscape metrics, is depicted in Fig 2’’ is a repetition of the definition of vegetation or land fragmentation.

**Response:** We have removed the repetitions and reordered the introduction section.

Line 121, Use Standard English grammar. It should be non- fragmented. Delete not fragmented.

**Response:** We have corrected it.

Why is it necessary to model and simulate vegetation or land fragmentation? What’s the societal and scientific relevance and contribution? Is not highlighted in the introduction section.

**Response:** We have respecified the need and contribution in the introduction section.

Line 146- delete overall methodology used standard terminology

**Response:** We have changed it to ‘research methodology and techniques’.

Line 155 -156, ‘‘before defining the models, Pearson correlation was used to exclude the multi-correlated and least-correlated factor’’ is not correct. Note that Pearson correlation a measure of the direction of relationship that exists between two continuous variables. I expected the authors to mention Correlation matrix or Principal Components Analysis in excluding the multi-correlated and least-correlated factor’ ’Correct this

**Response:** We have corrected it.

Line 161- Fig 4.should indicate that it’s a flowchart showing the steps or research methodology used in the study. Please revisit Fig 4 caption and better delete it ‘‘the overall method of the study‘‘is not catchy.

**Response:** We have changed it to ‘Steps of research methodology’.

The research methodology is too repetitive, confusing, long and winding and poorly written.

**Response:** We have removed the repetitions and We have employed a professional scientific editing service to enrich the content.

The discussion part of the manuscript is poorly written. It does not show depth. The discussion section is where you explore the underlying meaning of your research (ie modelling and simulating vegetation fragmentation or land fragmentation) by citing various sources, its possible implications in other areas of study, and the possible improvements that can be made in order to further develop the concerns of your research. How do you compare your results with those from other studies: Are they consistent? If not, discuss possible reasons for the difference. Discuss how could your findings be applied and extend the findings of previous studies?

**Response:** We have rewritten the discussion section including comparisons between prior studies and explained possible reasons.

**Reviewer #2**

The title

As the title of any paper should attract the reader and concisely reflects the actual content of the researchers’ contribution, I found the title of this manuscript needs some modification. For example, I suggest the following title “Modelling vegetation land fragmentation in urban areas of Western Province, Sri Lanka using an artificial intelligence-based simulation technique”

**Response:** We have changed the title.

The Abstract

While the abstract summaries the main idea of fragmentation as a landscape ecology term properly, several keys are missing from it mainly the research aims. Please directly state the main aim and objectives (or the research questions) of the study and write about the policy implications of the study area. Likewise, I suggest inserting a couple of sentences to mention all variables that have been incorporated in the modelling process.

**Response:** We have rewritten the abstract including the aim, objectives, and variables.

Introduction

Line 44 what does the number 392249 refer to and it seems that the citation is written in an integrated or standard way?

**Response:** We have corrected the errors in citations and references.

Line 62-63 the sentence needs to rewritten.

**Response:** We have rewritten it and have employed a professional scientific editing service to improve English.

Line 64 the authors have mentioned “studies point to….” while they have cited only one study (32).

**Response:** We have corrected it.

Overall, the introduction section involves both a theoretical framework and related work as well as introducing the main idea of the research. Nevertheless, it was NOT written in cohesion and logical flow style. Similarly, the analytical procedures, utilized spatial techniques for the analysis of land use land cover dynamics have not been made clear.

**Response:** We have strengthened the introduction section including spatial techniques. Also, we have restructured the introduction section.

Theoretical Framework

Besides the poor scientific literature about fragmentation of lands, spatial patterns and ecological process, the literature review section also lacks an adequate description of methods and analyses that previously used in studying several land use land cover LULC applications particularly outside the study area and region. I suggest, to expand the literature review by including additional references particularly the following:

- Spatial disparity patterns of green spaces and buildings in arid urban areas

- Cultivated Land Fragmentation and Its Influencing Factors Detection: A Case Study in Huaihe River Basin, China

- The evolution of urban sprawl: Evidence of spatial heterogeneity and increasing land fragmentation

**Response:** We have included the above studies and expanded the literature review by including additional references.

Other issues in this section should be resolved as follows:

o The authors should combine the two sections “the introduction and theoretical framework” into one section entitled “introduction and literature review”.

The authors should explain to the reader what motivated this research and why it is important to assess the vegetation land fragmentation in this area specifically.

o Similarly, and from a methodological perspective, various questions should be answered such as how the previous research analyzed and investigated vegetation land fragmentation in Sir Lanka and outside? What are the main spatial methods and techniques that have been used?

o The literature review is very poor and must be extended to include several studies that cover various issues that are relevant to this research.

**Response:** We have combined the two sections “the introduction and theoretical framework” into one section entitled “introduction and literature review”.

Methods

The title “method” should be changed into “material and methods”.

**Response:** We have corrected it.

I am wondering why the authors have not represented the identified parameters in a map. The authors should provide the readers with a map illustrates all spatial variables utilized in the analysis processes.

**Response:** We have included an additional Figure showing the spatial distribution of factors.

Line 187 the explanation of the equation’s symbols should be placed after NOT before.

**Response:** We have corrected it.

Findings

The authors should represent the spatial outputs of the modelling process. For instance, the reader expects to see one map for each category such as corridor, patch, and matrix.

The authors should add at least two maps for more clarity of the finding’s representation.

**Response:** Due to a lack of secondary data, this study investigated data under GNDs boundaries, which is the local level administrative boundary. This is one of the study's weaknesses. As a result, the results of this study cannot be interpreted in terms of corridor, patch, or matrix. Future research will, however, overcome this constraint. We've made it clear that there's a limitation.

Figure 12. “Rules of variables according to lekprofile algorithm” should be reproduced in a higher level of resolution.

**Response:** We have reproduced it in a higher level of resolution.

Overall, the results section lacks any sort of cause-and-effect relationships investigation. Consequently, the results have been written in methodological way and thus the authors must modify this section based on spatial patterns distribution across the study area.

**Response:** We have improved the figures indicating spatial references.

Discussion and conclusions

The discussion should tell the story of the science and answer the “why” question of the results.

**Response:** We have specified the reasons for the results.

The explanation is not adequately supported by pieces of evidence, reasoning, and thus, after carrying out the suggested analyses, a major revision is required for this section to clarify the discussion part and link it to the previous research.

**Response:** We have cited multiple studies to provide evidence and reasons for results. Also, we have provided suggestions for future studies.

The authors should mention the study’s limitations and drawbacks.

**Response:** We have specified the limitations in the discussion section and the summary in the conclusion section.

Many grammatical, stylistic and syntax errors are found across the manuscript.

**Response:** We have corrected grammatical, stylistic and syntax errors.
